# Editorial: Nutrition and sustainable development goal 15: life on land

**DOI:** 10.3389/fnut.2024.1453607

**Published:** 2024-07-17

**Authors:** Romanus Osabohien

**Affiliations:** ^1^Institute of Energy Policy and Research (IEPRe), Universiti Tenaga Nasional (UNITEN), Kajang, Malaysia; ^2^Publication Unit, DEPECOS Institutions and Development Research Centre (DIaDeRC), Ota, Nigeria

**Keywords:** environmental sustainability, food and nutrition security, life on land, sustainable agricultural practices, sustainable development, food supply chain management

## Introduction

Unarguably, nutrition sits at the heart of the United Nations (UN) Sustainable Development Goals (SDGs) (1–4). In addition to achieving “Zero Hunger” (SDG2), improvements in nutrition are critical to both achieve and reap the benefits of all seventeen goals. With good nutrition comes improved health and wellbeing (SDG3), enhanced educational and work productivity (SDGs 4 and 8), less poverty (SDG1) and reduced inequalities (SDGs 5 and 10). And with stronger and more sustainable environments, communities, and technologies (SDGs 6, 7, 9, 11–17) improved food security and nutrition will follow. As part of an innovative Research Topic showcasing nutrition in the context of the SDGs, this Research Topic focuses on Sustainable Development Goal 15: Life on Land.

Sustainable Development Goal 15: Life on Land stands as a beacon of hope for the conservation and sustainable management of terrestrial ecosystems, essential for the wellbeing of both the planet and its inhabitants (5, 6). As we navigate the intricate web of global challenges, the intersection of nutrition and environmental sustainability emerges as a crucial point of convergence (6, 7). The choices we make in food production, consumption, and land use have far-reaching implications for biodiversity, ecosystem health, and human nutrition (3, 5).

In this Research Topic, we delve into the intricate relationship between nutrition and Sustainable Development Goal 15, exploring how dietary choices impact the delicate balance of life on land. By understanding the interconnectedness of nutrition and biodiversity, we can unlock new pathways toward a more sustainable future where human health and environmental conservation go hand in hand.

## Summary of studies in the Research Topic

Eight articles were published in this Research Topic. The first article, by Li et al. investigated the effects of substituting dietary corn with *Hypsizygus marmoreus* mushroom stem waste (HSW) in the diet of geese and the results indicate that 24% HSW substitution of corn could improve goose serum ALB and fat metabolism, and increase serum antioxidant capacity.

The second article, Ngigi et al. documented existing knowledge on traditional fruits, vegetables and pulses in Kenya and Ethiopia. The aimed was to identify neglected and underutilized species with high potential for food security, for their economic value and contribution to sustainable agriculture, based on a literature review and confirmation of existing data by local experts. The study recommended that since women play a central role in traditional food systems, their empowerment, and hence resilience, increase the positive impact they can have on the households' dietary diversity.

In the third article, Ismail et al. results show that based on the chemical composition, *Polycladia myrica* was the most valuable species, with a comparatively high protein content of 22.54%, lipid content of 5.21%, fucoxanthin content of 3.12 μg/g, β-carotene content of 0.55 mg/100 g, and carbohydrate content of 45.2%. The fourth study, Otu et al. examined how learning from One-Health approaches to explore links between farming practices, animal, human and ecosystem health in Nigeria.

In the fifth article, Khan et al. findings indicated the fern preference for middle elevation zones with high organic matter and acidic to neutral soil (pH ≥ 6.99) for retaining higher nutritional contents. In the sixth article, Ranieri et al. selected 34 vegetables for continuous cultivation and provisions to the school kitchens and found that Nine tons of vegetables were produced and provided to 90 municipal schools from 2020 to 2023. The study finds that leafy vegetables accounted for most the production, with a total weight of 6,441 kg corresponding to 71.6% of the total harvest. Kitchen teams were trained throughout the project duration.

In the seventh article, Kals et al. study determined whether dried BSFL or dried CN could partly substitute the commercial diet when growing Nile tilapia (*Oreochromis niloticus*) in a smallholder farm aggregated in an aquapark. The nutritional values and cost-effectiveness of the alternative feeding strategies were compared to commercial diet (CD) only. During an 84-day experimental period, Nile tilapia were fed one of the three feeding strategies, including the use of only the commercial diet, to be compared with diets replacing 20% of the commercial diet with BSFL or CN. In the eighth article, Fan et al. concluded that unreasonable tillage practices destroy the structure of the ploughed layer and affect soil physical parameters.

## Conclusion

This Research Topic discusses the intricate relationship between nutrition and Sustainable Development Goal 15, which focuses on protecting life on land and promoting sustainable land use practices. It highlights the importance of biodiversity in ensuring food security, nutrition, and overall human well-being. Articles in this Research Topic emphasize the need to adopt sustainable agricultural practices that preserve biodiversity, promote healthy diets, and mitigate the negative impacts of climate change on food systems. It also underscores the role of individuals, communities, and policymakers in promoting sustainable food production and consumption to achieve the objectives of SDG 15.

In conclusion, the articles published in the Research Topic emphasize the critical link between nutrition, biodiversity, and Sustainable Development Goal 15. It underscores the urgency of addressing challenges such as food insecurity, malnutrition, and environmental degradation to ensure a sustainable future for all. By promoting sustainable land use practices, preserving biodiversity, and adopting healthy diets, we can contribute to achieving the goals of SDG 15 and safeguarding life on land for present and future generations. It calls for collective action and collaboration among stakeholders to address these complex issues and create a more sustainable and resilient food system that benefits both people and the planet.

## Author contributions

RO: Conceptualization, Data curation, Formal analysis, Investigation, Methodology, Supervision, Writing – original draft, Writing – review & editing.
